# New targets of morphine postconditioning protection of the myocardium in ischemia/reperfusion injury: Involvement of HSP90/Akt and C5a/NF-κB

**DOI:** 10.1515/med-2021-0340

**Published:** 2021-10-18

**Authors:** Rong-Hui Tu, Dong-Xiao Wang, Guo-Qiang Zhong, Jian-Jun Meng, Hong Wen, Jie-Feng, Qi Bi, Yan He

**Affiliations:** Department of Geriatric Cardiology, First Affiliated Hospital, Guangxi Medical University, 6 Shuangyong Road, Nanning, 530021, Guangxi, China; Guangxi Key Laboratory of Precision Medicine in Cardio-Cerebrovascular Diseases Control and Prevention, First Affiliated Hospital, Guangxi Medical University, Nanning, 530021, Guangxi, China; Guang Xi Clinical Research Center for Cardio-Cerebrovascular Diseases, First Affiliated Hospital, Guangxi Medical University, Nanning, 530021, Guangxi, China; Department of Cardiology, First Affiliated Hospital, Guangxi Medical University, Nanning, 530021, China; Departments of Geriatric Health Care Center, First Affiliated Hospital, Guangxi Medical University, Nanning, 530021, Guangxi, China; School of Pharmaceutical Science, Guangxi Medical University, Nanning, 530021, Guangxi, China

**Keywords:** morphine postconditioning, HSP90, Akt, C5a, NF-κB

## Abstract

**Background:**

Activation of the complement component 5a (C5a) and nuclear factor κB (NF-κB) signaling is an important feature of myocardial ischemia/reperfusion (I/R) injury and recent studies show that morphine postconditioning (MP) attenuates the myocardial injury. However, the mediating cardioprotective mechanisms remain unclear. The present study explores the role and interaction of heat shock protein 90 (HSP90), Akt, C5a, and NF-κB in MP-induced cardioprotection.

**Methods:**

Male Sprague Dawley rats (*n* = 160) were randomized into eight groups (*n* = 20 per group). Rats in the sham group underwent thoracotomy, passing the ligature through the heart but without tying it (150 min), and the other seven groups were subjected to 30 min of anterior descending coronary artery occlusion followed by 2 h of reperfusion and the following treatments: I/R (30 min of ischemia and followed by 2 h of reperfusion); ischemic postconditioning (IPostC, 30 s of ischemia altered with 30 s of reperfusion, repeated for three cycles, and followed by reperfusion for 2 h); MP (0.3 mg/kg morphine administration 10 min before reperfusion); MP combined with the HSP90 inhibitor geldanamycin (GA, 1 mg/kg); MP combined with the Akt inhibitor GSK-690693 (GSK, 20 mg/kg); and MP combined with the C5a inhibitor PMX205 (PMX, 1 mg/kg/day, administration via drinking water for 28 days) and MP combined with the NF-κB inhibitor EVP4593 (QNZ, 1 mg/kg). All inhibitors were administered 10 min before morphine and followed by 2 h reperfusion.

**Results:**

MP significantly reduced the I/R-induced infarct size, the apoptosis, and the release of cardiac troponin I, lactate dehydrogenase (LDH), and creatine kinase-MB. These beneficial effects were accompanied by increased expression of HSP90 and p-Akt, and decreased expression of C5a, NF-κB, tumor necrosis factor α, interleukin-1β, and intercellular cell adhesion molecule 1. However, HSP90 inhibitor GA or Akt inhibitor GSK increased the expression of C5a and NF-κB and prevented MP-induced cardioprotection. Furthermore, GA inhibited the MP-induced upregulation of p-Akt, while GSK did not affect HSP90, indicating that p-Akt acts downstream of HSP90 in MP-induced cardioprotection. In addition, C5a inhibitor PMX enhanced the MP-induced downregulation of NF-κB, while NF-κB inhibitor QNZ had no effect on C5a, indicating that the C5a/NF-κB signaling pathway is involved in MP-induced cardioprotection.

**Conclusion:**

HSP90 is critical for MP-mediated cardioprotection possibly by promoting the phosphorylation of Akt and inhibiting the activation of C5a and NF-κB signaling and the subsequent myocardial inflammation, ultimately attenuating the infarct size and cardiomyocyte apoptosis.

## 1 Introduction

Myocardial ischemia/reperfusion (I/R) injury can be extremely severe or even fatal, reducing remarkably the benefits of revascularization in acute myocardial infarction. Increasing evidence shows that opioid receptors are an emerging therapeutic target in I/R injury and cardioprotection [1,2]. Recent clinical trials using opioid receptor agonists during coronary artery bypass surgery have yielded encouraging results in reducing myocardial injury [3]. Furthermore, morphine was reported to enhance the cardioprotection of remote conditioning in patients with percutaneous coronary intervention [4]. However, the cardioprotective mechanisms of morphine in I/R injury have not been completely elucidated.

Heat shock protein 90 (HSP90) is a cellular stress protein that plays a critical role in ischemic preconditioning and postconditioning in cardioprotection [5,6,7]. Recently, a role for HSP90 in morphine-induced cardioprotection was reported [8]. In addition, as a conserved molecular chaperone, HSP90 is critical for the integrity and function of its signaling client proteins. Akt, a client protein of HSP90, is activated by morphine and plays an important role in the regulation of cellular growth [9]. *In vivo* studies demonstrated that hydromorphine postconditioning reduced myocardial I/R injury by activating the Akt pathway [10]. However, the interaction between HSP90 and Akt in morphine-induced cardioprotection and the corresponding details are unclear.

Complement activation, which culminates in an inflammatory response, plays a predominant role in myocardial I/R injury [11]. The complement system consists of more than 30 plasma and cell membrane proteins and is responsible for recognizing hypoxia or ischemia signals. During myocardial I/R, particularly in the early stages, the rapid activation of complement products C3 and component 5a (C5a) induces cardiomyocytes apoptosis or necrosis through inflammatory cell infiltration and cytokine release [12]. The excessive generation of C3 and C5a further activates nuclear factor κB (NF-κB) leading to intensified inflammation [13,14,15]. Conversely, inhibition of the complement cascade and the NF-κB signaling significantly attenuates I/R injury [16,17]. However, it is not clear whether morphine-induced cardioprotection is responsible for the inhibition of the complement cascade and the NF-κB signaling. Only a few investigations have been conducted to determine the role of HSP90/Akt interaction with C5a/NF-κB in morphine-induced cardioprotection.

In the present study, we tested the involvement of HSP90/Akt in the inhibition of C5a/NF-κB through the use of specific inhibitors and the consequent inflammatory responses in morphine postconditioning (MP). A rat model of myocardial I/R injury was established to examine the effects of MP on infarct size; cardiomyocyte apoptosis; and release of cardiac troponin I (cTnI), lactate dehydrogenase (LDH), and creatine kinase-MB (CK-MB) in plasma. The protein levels of HSP90, phoso-Akt (p-Akt), C5a, and NF-κB were examined by Western blot analysis. The mRNA levels of tumor necrosis factor α (TNF-α), interleukin (IL)-1β, and intercellular cell adhesion molecule 1 (ICAM-1) were examined. The effects of HSP90 inhibitor geldanamycin (GA), Akt inhibitor GSK-690693 (GSK), C5a inhibitor PMX 205 (PMX), and NF-κB inhibitor EVP4593 (QNZ) on MP were also investigated.

## 2 Materials and methods

### 2.1 Animals

Male adult Sprague Dawley rats, weighing 250 ± 20 g, were obtained from the experimental animal center of Guangxi Medical University, China. The rats were housed in ventilated cages under standard laboratory conditions (temperature: 25 ± 2°C; humidity: 50 ± 15%; photoperiod: 12 h light/dark cycle photoperiod). The rats had *ad libitum* access to food and water.


**Ethics approval and consent to participate:** The experimental protocols were performed in strict accordance with the National Institutes of Health Guide for the Care and Use of Laboratory Animals and were approved by the Animal Protection and Use Committee of Guangxi Medical University.
**Patient consent for publication:** Not applicable.

### 2.2 Myocardial I/R model

The rats were anesthetized with sodium pentobarbital (50 mg/kg, IP) and connected to a rodent ventilator at a respiratory rate of 80 cycles/min and a tidal volume of 10 mL/kg body weight. The chest was opened over the fifth intercostal space on the left to expose the heart. A 6-0 suture was passed through the left anterior descending coronary artery and then through a small flexible vinyl tube. The suture was ligated for 30 min to induce myocardial ischemia, and then untied to allow reperfusion for 2 h. Elevated ST segments in the electrocardiogram and the myocardial blanching of the ligated blood supply area served as indications of successful model construction. At the end of the reperfusion, the rats were euthanized. A portion of the anterior wall of the left ventricle myocardium near the apex of the heart and blood samples were obtained for further analyses.

### 2.3 Experimental groups

The rats were randomized into eight groups (*n* = 20 per group). (1) Sham group: rats underwent thoracotomy, passing the ligature through the heart but without tying it (150 min). (2) I/R group: 30 min of ischemia and followed by 2 h of reperfusion. (3) IPostC group: 30 s of ischemia altered with 30 s of reperfusion, repeated for three cycles, and followed by reperfusion for 2 h. (4) MP group: I/R followed by morphine administration (0.3 mg/kg [18] IV bolus; Sigma-Aldrich, St. Louis, MO, USA) 10 min before the beginning of reperfusion and then reperfused for 2 h. (5) MP + GA group: administration of the HSP90 inhibitor geldanamycin (GA, 1 mg/kg [19] IP; Sigma-Aldrich). (6) MP + GSK group: administration of the Akt inhibitor GSK-690693 (GSK, 20 mg/kg [20] IP; Med Chem Express, NJ, USA). (7) MP + PMX group: administration of the C5a inhibitor-PMX 205 (PMX, 1 mg/kg/day [21], administration for 28 days via drinking water; Med Chem Express, NJ, USA). (8) MP + QNZ group: administration of the NF-κB inhibitor EVP4593 (QNZ, 1 mg/kg [22]; IP, Selleck, USA). All inhibitors were administered 10 min before morphine and followed by 2 h reperfusion.

### 2.4 Myocardial infarction size analysis

Separate experiments were performed to determine the infarction size (*n* = 5 for each group). At the end of reperfusion, the left anterior descending coronary artery was retightened and 2% Evans blue dye was injected into the inferior vena cava and washed with PBS buffer. The stained heart was then frozen and cut into 2 mm thick slices. Then the slices were incubated in 1% 2,3,5-triphenyltetrazolium chloride (TTC; Sigma-Aldrich) at 37°C for 15 min to delineate the size of the infarction. The infarcted regions were quantified using the digital imaging software Image-Pro Plus version 6.0 (Media Cybernetics, Bethesda, MD). The damage was calculated as a percentage of the infarct size over the left ventricle (IS/LV).

### 2.5 Biochemical analysis

Before removing the heart, blood samples of 5 ml were collected and centrifuged at 3,000 g for 5 min to obtain the serum. The levels in serum of CK-MB, LDH, and cTnI were determined using an automatic biochemical analyzer (IDEXX Laboratories Inc., Maine, USA).

### 2.6 Terminal deoxynucleotidyl transferase-mediated dUTP-X nick end labeling (TUNEL) staining

In brief, paraformaldehyde-fixed heart tissue blocks were incubated with proteinase K, then washed, dehydrated, embedded in paraffin, and sectioned at 5 μm using a microtome. Apoptotic cells were identified using a TUNEL detection kit (Roche Diagnostics, Basel, Switzerland), following the manufacturer’s protocol. The stained cells were observed using a microscope, in at least five randomly chosen fields with apoptotic cells. The nuclei with blue staining were labeled as normal cardiomyocytes. Nuclei with brown staining were labeled as TUNEL-positive cells. The apoptotic index was calculated as the number of TUNEL-positive cells/total number of cardiomyocytes × 100%.

### 2.7 Quantitative reverse transcriptase-polymerase chain reaction

Total RNA was extracted from heart tissues and purified using the Trizol reagent (Invitrogen, CA, USA). The cDNA was synthesized using a PrimeScript™ RT reagent Kit (Takara Bionic, Otsu, Japan), and quantitative reverse transcriptase-polymerase chain reaction (qPCR) was performed using SYBR standard qPCR mix (Takara Bionic) in an ABI Prism 7500 System (Thermo Fisher Scientific, Shanghai, China) according to the manufacturer’s instruction. Glyceraldehyde-3-phosphate dehydrogenase was used as the housekeeping gene. The relative mRNA expression was analyzed using the 2^−ΔΔCt^ method. The following primers were used for the detection of IL-1β forward 5′-ATCTCACAGCAGCATCTCGACAAG-3′ and reverse 5′-CACACTAGCAGGTCGTCATCATCC-3′, TNF-α forward 5′-GCATGATCCGAGATGTGGAACTGG-3′ and reverse 5′-CGCCACGAGCAGGAATGAGAAG-3′, ICAM-1 forward 5′-TGTCGGTGCTCAGGTATCCATCC-3′ and reverse 5′-TTCGCAAGAGGAAGAGCAGTTCAC-3′.

### 2.8 Western blot

Myocardial tissues from each group were homogenized using radioimmunoprecipitation assay lysis buffer (Solarbio, Beijing, China) with an ultrasonic grinder, and then placed on ice for 30 min to dissolve the tissues. Subsequently, a 2 mL centrifuge tube was centrifuged at 12,000 g for 15 min at 4°C, and the supernatant was collected and the protein concentration was determined using an enhanced BCA protein assay kit (Beyotime, Shanghai, China). Then, 10 μL of protein per sample were loaded and separated by 10% SDS-PAGE and then transferred onto the PVDF membranes (Millipore, Bedford, MA, USA). The membranes were blocked with 5% BSA for 1 h at 25°C, and then incubated with the primary antibodies (anti-HSP90, 1:5,000, Proteintech, Chicago, USA; anti-p-Akt, 1:5,000, Proteintech; anti-β-actin, 1:5,000, Proteintech; anti-C5a, 1:5,000, Invitrogen; anti- NF-κB p65, 1:1,000, Invitrogen) at 4°C for 24 h. Then the membranes were thoroughly washed using TBST and incubated with the secondary antibody, an HRP-labeled goat anti-rabbit IgG (1:12,000) at 25°C for 1 h. The ECL Plus detection system (Millipore Corporation, Billerica, MA, USA) was used for the detection of band immune-detection according to the manufacturer’s instructions.

### 2.9 Statistical analysis

Data were analyzed using the statistical analysis software SPSS 23.0 (SPSS, Inc, Chicago, IL, USA). Measured data are expressed as mean ± standard deviation. Multiple group means were compared using a one-way analysis of variance, and the least significant difference method was used for comparison between groups. *P* < 0.05 was considered statistically significant.

## 3 Results

In this study, 160 rats were used. Due to a technical failure of the ventilator and the cardiogenic shock during reperfusion, four rats died and were subsequently excluded from further analysis: one belonged to the IPostC group, one to the I/R group, one to the MP + GA group, and one to the MP + GSK group. The results presented correspond to the remaining 156 rats.

### 3.1 MP diminished the I/R-induced myocardial infarct size

**Figure 1:**
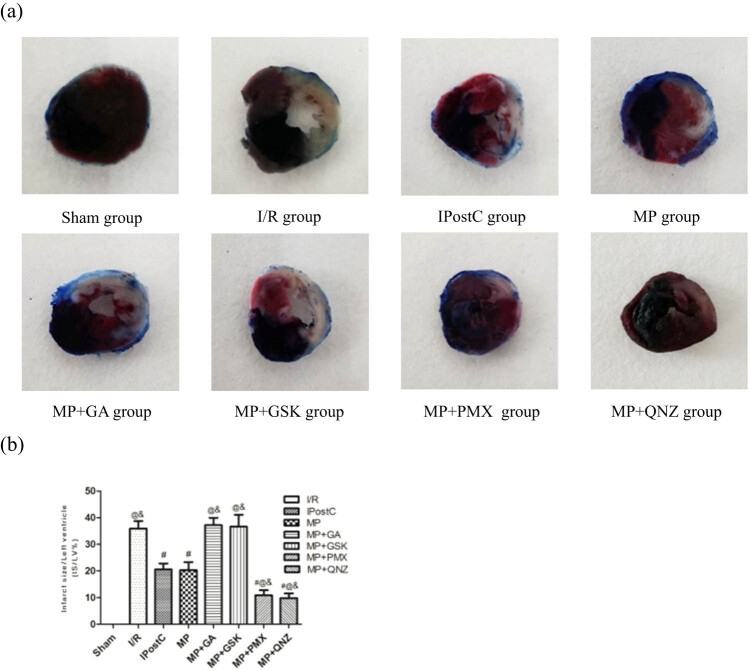
(a) Effects of IPostC, MP, and MP with treatment with the GA, GSK, PMX, and QNZ on myocardial infarct size after cardiac I/R injury (IS/LV). (b) Quantitative analysis of myocardial infarct size. The results presented the mean ± standard deviation. ^#^ *P* < 0.05 vs I/R group; ^@^ *P* < 0.05 vs IPostC group; ^&^ *P* < 0.05 vs MP group, *n* = 5 for each group. I/R: ischemia/reperfusion; MP: morphine postconditioning; GA: geldanamycin; GSK: GSK-690693; PMX: PMX 205; QNZ: EVP4593.

After 2 h reperfusion, the IS/LV was 35.93 ± 2.81% in the I/R group. In the IPostC group and the MP group, it significantly decreased compared to the I/R group to 20.59 ± 2.21% and 20.33 ± 2.96%, respectively (all *P* < 0.05, Figure 1). No significant differences were observed between the IPostC group and the MP group. However, the protective effects induced by MP were reversed by the administration of GA (37.22 ± 2.77%) and GSK (36.65 ± 4.46%). Both PMX and QNZ strengthened the MP-induced cardioprotection (10.88 ± 1.92% and 9.83 ± 1.78%, respectively).

### 3.2 MP attenuated I/R-induced apoptosis

**Figure 2:**
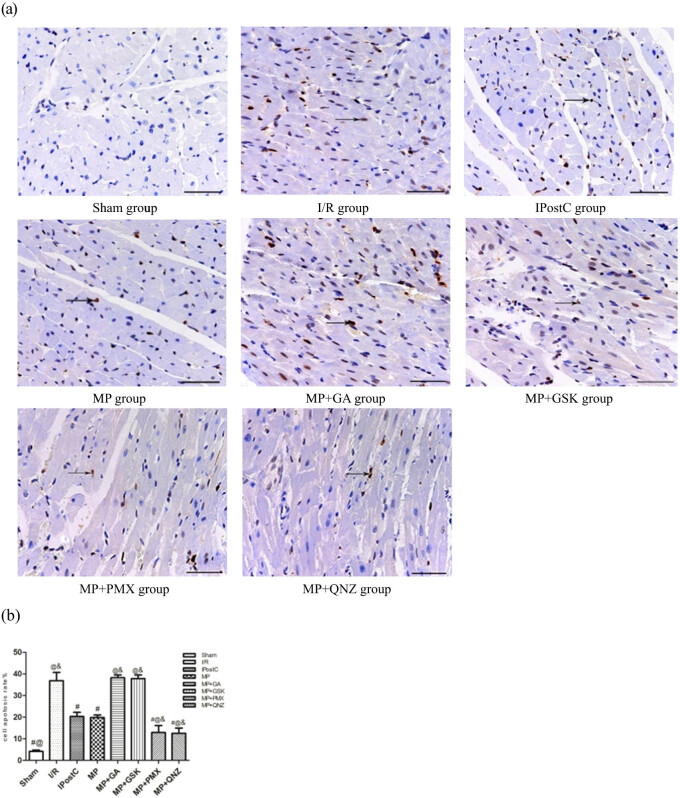
(a) Effects of IPostC, MP, and MP with treatment with the GA, GSK, PMX, and QNZ on apoptosis after myocardial I/R. (b) Quantitative analysis of cell apoptotic rate, results presented in a bar graph are mean ± standard deviation. Apoptotic cardiomyocyte nuclei appear brown stained, whereas TUNEL-negative nuclei appear blue. Arrow indicates TUNEL-positive cells. TUNEL stain ×400, all bars = 50 μm. ^#^ *P* < 0.05 vs I/R group; ^@^ *P* < 0.05 vs IPostC group; ^&^ *P* < 0.05 vs MP group, *n* = 5 for each group. I/R: ischemia/reperfusion; MP: morphine postconditioning; GA: geldanamycin; GSK: GSK-690693; PMX: PMX 205; QNZ: EVP4593.

There were numerous apoptotic cells in the I/R group (36.84 ± 3.87%). In the IPostC group and the MP group, apoptosis levels significantly decreased reaching 20.31 ± 1.92% and 19.87 ± 1.13%, respectively, when compared with the I/R group (all *P* < 0.05, Figure 2). No significant differences were observed between the IPostC group and the MP group. However, the anti-apoptotic and cardiomyocyte-limiting effects of MP were blocked by GA (38.25 ± 1.26%) and GSK (37.80 ± 1.72%). Both PMX and QNZ enhanced the effect of MP (12.94 ± 3.16% and 12.51 ± 2.46%, respectively).

### 3.3 MP attenuated I/R-induced myocardial injury

Serum CK-MB, LDH, and cTnI levels were significantly higher in the I/R group than in the sham group (Table 1). Compared to the I/R group, the IPostC group had significantly lower levels of CK-MB (1384.40 ± 265.71 U/L vs 890.80 ± 136.81 U/L), LDH (1465.6 ± 269.48 U/L vs 1033.60 ± 95.90 U/L), and cTnI (23.71 ± 3.55 ng/mL vs 13.11 ± 1.03 ng/mL) after I/R. Following treatment with morphine, the levels of CK-MB (942.60 ± 40.70 U/L), LDH (1044.60 ± 103.69 U/L), and cTnI (12.12 ± 1.44 ng/mL) were similar to the IPostC group’s levels. The effect of MP was strengthened by PMX and QNZ administration. There were no significant differences between MP + GA, MP + GSK, and the I/R group (Table 1).

**Table 1:** Levels of CK-MB, LDH, and cTnI in serum

Groups	CK-MB (U/L)	LDH (U/L)	cTnI (ng/mL)
Sham	434.20 ± 26.89^#@^	458.80 ± 22.24^#@^	0.96 ± 0.88^#@^
I/R	1384.40 ± 265.71^@&^	1465.60 ± 269.48^@&^	23.71 ± 3.55^@&^
IPostC	890.80 ± 136.81^#^	1033.60 ± 95.90^#^	13.11 ± 1.03^#^
MP	942.60 ± 40.70^#^	1044.60 ± 103.69^#^	12.12 ± 1.44^#^
MP + GA	1266.40 ± 220.84^@&^	1434.60 ± 189.32^@&^	21.87 ± 2.05^@&^
MP + GSK	1385.80 ± 276.20^@&^	1415.40 ± 255.58^@&^	21.78 ± 1.37^@&^
MP + PMX	661.00 ± 42.43^#@&^	775.40 ± 30.57^#@&^	5.80 ± 0.59^#@&^
MP + QNZ	654.60 ± 43.85^#@&^	774.60 ± 49.98^#@&^	5.58 ± 0.56^#@&^

Data are presented as mean ± standard deviation. ^#^ *P* < 0.05 vs I/R group; ^@^ *P* < 0.05 vs IPostC group; ^&^ *P* < 0.05 vs MP group, *n* = 5 for each group. I/R: ischemia/reperfusion; MP: morphine postconditioning; GA: geldanamycin; GSK: GSK-690693; PMX: PMX 205; QNZ: EVP4593.

### 3.4 MP attenuated myocardial I/R injury via the HSP90/Akt pathway

**Figure 3:**
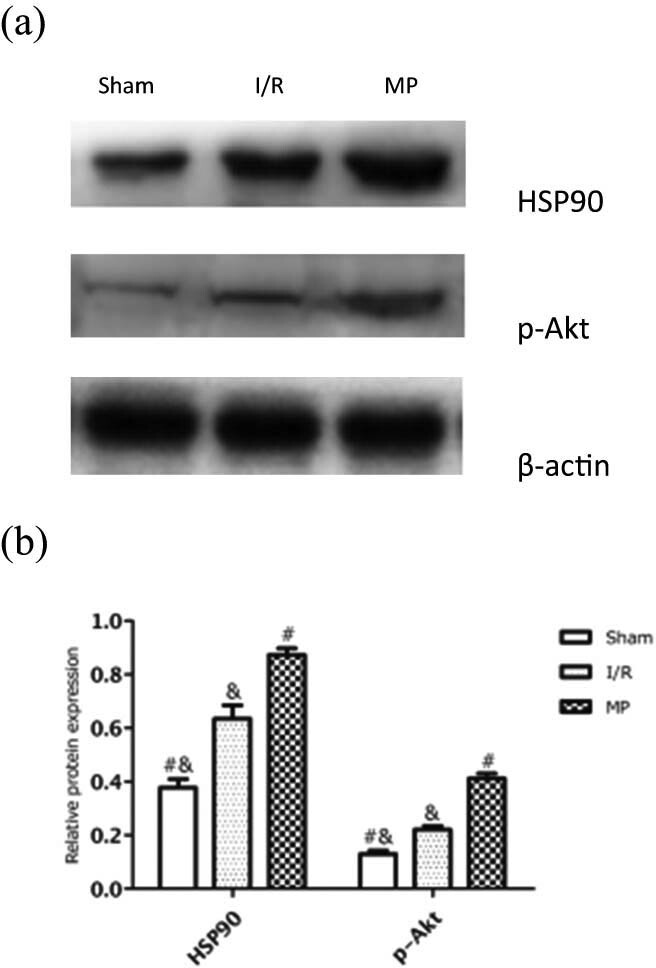
Effects of MP on HSP90 and p-Akt protein expression. (a) Representative Western blots showing the expressions of HSP90 and p-Akt. (b) Relative expression of HSP90 and p-Akt proteins. Values are presented as mean ± standard deviation. ^#^ *P* < 0.05 vs I/R group; ^&^ *P* < 0.05 vs MP group; *n* = 5 for each group. I/R: ischemia/reperfusion; MP: morphine postconditioning.

**Figure 4:**
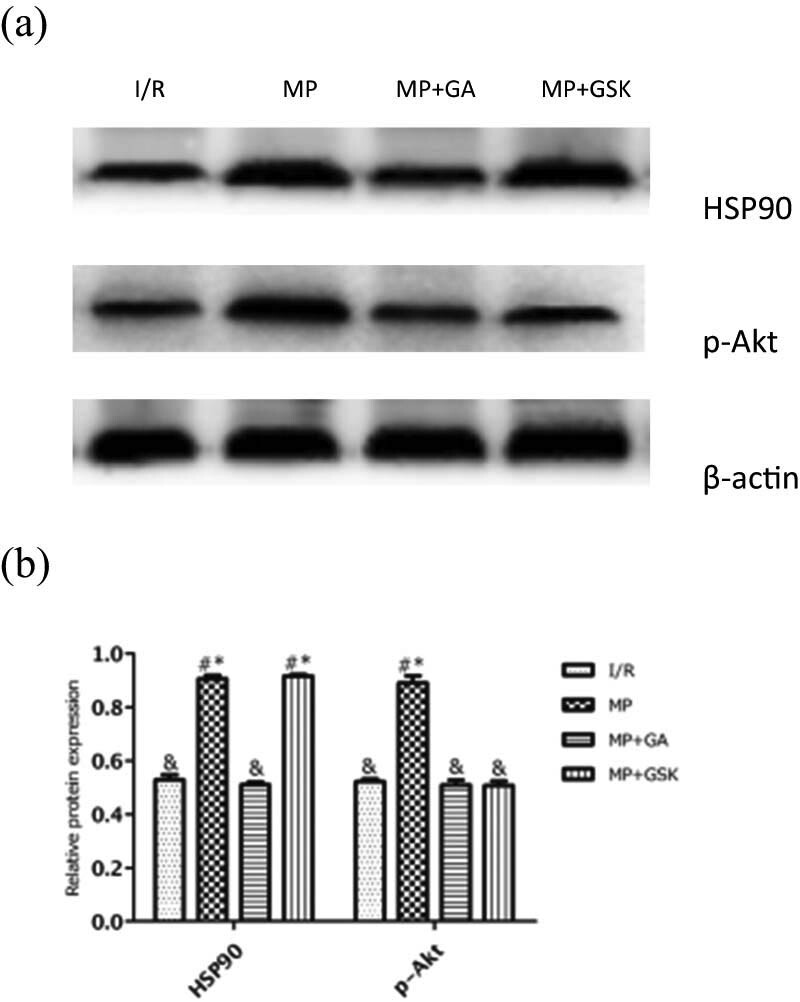
Effects of GA and GSK on HSP90 and p-Akt protein expression. (a) Representative Western blots showing the expressions of HSP90 and p-Akt. (b) Relative expression of HSP90 and p-Akt proteins. Values are presented as mean ± standard deviation. ^#^ *P* < 0.05 vs I/R group; ^&^ *P* < 0.05 vs MP group; ^*^ *P* < 0.05 vs MP + GA group, *n* = 5 for each group. I/R: ischemia/reperfusion; MP: morphine postconditioning; GA: geldanamycin; GSK: GSK-690693.

To determine whether HSP90 and Akt were involved in MP, we first examined the protein expression of HSP90 and p-Akt in myocardia. As shown in Figure 3, MP significantly increased the protein levels of HSP90 and p-Akt compared with the I/R group (87.36 ± 2.44% vs 63.47 ± 4.95% and 41.19 ± 1.84% vs 22.01 ± 1.45%, respectively; *P* < 0.05). This suggests that MP-induced cardioprotection was related to HSP90 and p-Akt, for which we further studied the interaction between HSP90 and p-Akt in MP by inhibiting HSP90 and Akt activity with the HSP90 inhibitor GA and the Akt inhibitor GSK, respectively. As shown in Figure 4, GA administration attenuated the expression of HSP90 and p-Akt compared with the MP group (51.02 ± 1.11% vs 90.67 ± 1.35% and 50.96 ± 1.80% vs 89.14 ± 2.65%, respectively; *P* < 0.05), while GSK administration attenuated the expression of p-Akt (50.76 ± 1.66% vs 89.14 ± 2.65%) but not HSP90 (91.73 ± 0.63% vs 90.67 ± 1.35%). These results indicated that HSP90, upstream of p-Akt, mediates the morphine-induced cardioprotection.

### 3.5 MP attenuated myocardial I/R injury by inhibiting the C5a/NF-κB pathway and the expression of pro-inflammatory cytokines

**Figure 5:**
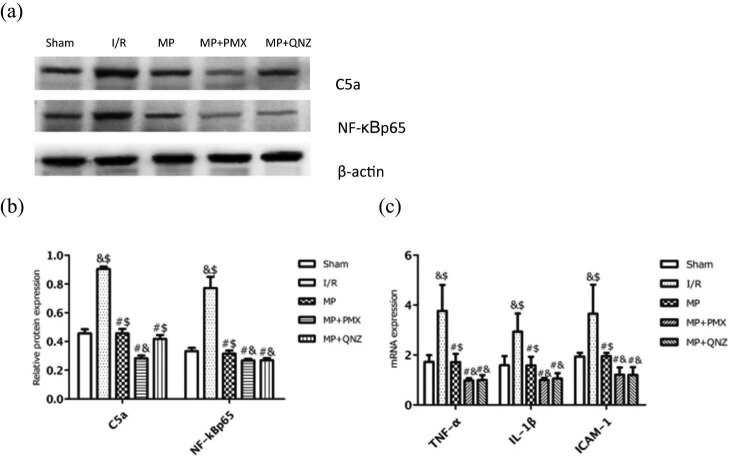
Effects of PMX and QNZ on C5a, NF-κBp65, TNF-α, IL-1β, and ICAM-1 expression. (a) Representative Western blots showing the expression of C5a and NF-κBp65. (b) Relative expression of C5a and NF-κBp65 proteins. (c) The mRNA levels of TNF-α, IL-1β, and ICAM-1 were measured using qPCR in different groups. Values are presented as mean ± standard deviation. ^#^ *P* < 0.05 vs I/R group; ^&^ *P* < 0.05 vs MP group; ^$^ *P* < 0.05 vs MP + *P*MX group; *n* = 5 for each group. I/R: ischemia/reperfusion; MP: morphine postconditioning; GA: geldanamycin; GSK: GSK-690693; PMX: PMX 205; QNZ: EVP4593.

To further assess the effect of MP on complement inhibition, we examined the protein expression of C5a and NF-κB, and measured the mRNA levels of the pro-inflammatory cytokines, including TNF-α, IL-1β, and ICAM-1. As shown in Figure 5a and b, MP led to significantly diminished protein levels of C5a (90.58 ± 1.53% vs 45.58 ± 2.30%) and NF-κB (77.20 ± 7.88% vs 31.51 ± 2.22%) compared to the I/R group. Likewise, the mRNA levels of TNF-α, IL-1β, and ICAM-1 were significantly decreased (Figure 5c; all *P* < 0.05), suggesting that inhibiting C5a and NF-κB results in morphine-induced cardioprotection. We also studied the interaction between C5a and NF-κB in MP using the C5a inhibitor PMX and the NF-κB inhibitor QNZ. As shown in Figure 5a and b, PMX administration further decreased expression of C5a (28.33 ± 1.92% vs 45.58 ± 2.30%) and NF-κB (26.83 ± 1.17% vs 31.51 ± 2.22%) compared with the MP group, while QNZ administration decreased even more the expression of NF-κB (26.84 ± 1.64% vs 31.51 ± 2.22%) but not C5a (41.90 ± 2.56% vs 45.58 ± 2.30%). These anti-inflammatory effects were reinforced when either PMX or QNZ was administered, suggesting that MP blocked myocardial inflammatory response by inhibiting C5a or NF-κB, subsequently reducing the release of the proinflammatory cytokines including TNF-α, IL-1β, and ICAM-1. These results suggest that morphine-induced cardioprotection is mediated by the inhibition of C5a/NF-κB.

### 3.6 MP inhibited C5a/NF-κB via HSP90/Akt pathway

**Figure 6:**
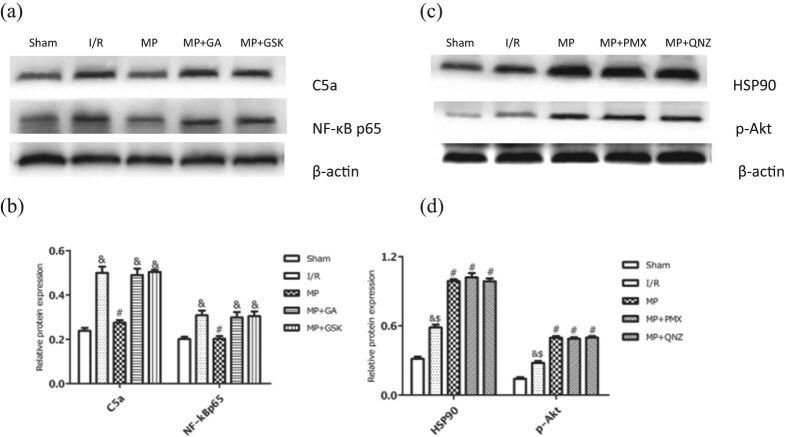
Effects of GA and GSK on C5a and NF-κBp65 protein expression. (a) Representative Western blots showing the expression of C5a and NF-κBp65. (b) Relative expression of C5a and NF-κBp65 proteins. (c) Representative Western blots showing the expression of HSP90 and p-Akt. (d) Relative expression of HSP90 and p-Akt proteins. Values are presented as mean ± standard deviation. ^#^ *P* < 0.05 vs I/R group; ^&^ *P* < 0.05 vs MP group; ^$^ *P* > 0.05 vs MP + *P*MX group; *n* = 5 for each group. I/R: ischemia/reperfusion; MP: morphine postconditioning; GA: geldanamycin; GSK: GSK-690693; PMX: PMX 205; QNZ: EVP4593.

Next, we evaluated the HSP90/Akt-pathway involvement in inhibiting C5a/NF-κB in MP. As shown in Figure 6a and b, both the HSP90 inhibitor GA and the Akt inhibitor GSK significantly increased the expression of C5a (48.94 ± 2.95% vs 27.45 ± 1.19%, 50.30 ± 1.01% vs 27.45 ± 1.19%, respectively) and NF-κB (29.80 ± 2.46% vs 20.26 ± 1.16%, 30.41 ± 2.16% vs 20.26 ± 1.16%, respectively) compared with the MP group. That means that GA and GSK blocked the MP-induced suppression of C5a/NF-κB, highlighting the role of HSP90/Akt in MP-induced cardioprotection through inhibiting C5a and NF-κB. On the contrary, neither the C5a inhibitor PMX nor the NF-κB inhibitor QNZ had any effect on the expression of HSP90 (101.93 ± 3.69% vs 90.44 ± 2.11%, 98.63 ± 2.39% vs 90.44 ± 2.11%, respectively; Figure 6c and d) and Akt (48.83 ± 1.33% vs 49.70 ± 1.46%, 49.84 ± 1.31% vs 49.70 ± 1.46%, respectively; Figure 6c and d) when compared with the MP group’s levels. Taken together, these results indicated that the HSP90/Akt pathway is involved in the cardioprotection of MP through the inhibition of the C5a/NF-κB signaling pathway.

## 4 Discussion

The key finding of this study is that MP reduced I/R-induced myocardial injury by inhibiting C5a and NF-κB signaling in an HSP90/Akt-dependent manner. We showed that MP significantly decreased the infarct size, the apoptosis, and diminished the levels of released CK-MB, LDH, and cTnI. These beneficial effects were accompanied by decreased expression of C5a, NF-κB, TNF-α, IL-1β, and ICAM-1, and increased expression of HSP90 and p-Akt. Therefore, the results of this study demonstrated for the first time that HSP90 is critical for MP-mediated cardioprotection possibly by promoting the phosphorylation of Akt and inhibiting the activation of C5a and NF-κB. This subsequently attenuates the myocardial inflammation and ultimately leads to a reduction in infarct size and apoptosis. Our data reveal a novel mechanism of MP protection.

Myocardial reperfusion injury and its therapy are under research in cardiology. Nowadays, there is increasing evidence that opioid receptors are an emerging therapeutic target in I/R injury and cardioprotection [23,24]. Small clinical studies using opioid receptor agonists in the reperfusion setting during coronary artery bypass surgery demonstrated beneficial effects in reducing myocardial injury [3]. Furthermore, morphine enhances the cardioprotection of remote conditioning to patients undergoing percutaneous coronary intervention [4]. Here we used an *in vivo* rat model of I/R to mimic clinical myocardial infarction and to explore the effects and possible mechanisms of morphine in myocardial reperfusion injury. We found that morphine significantly decreased the infarct size; cardiomyocyte apoptosis; and the release of CK-MB, LDH, and cTnI. Suppressing the HSP90 function with GA prevented the morphine-induced cardioprotection. These findings support a significant role for HSP90 in MP.

HSP90 is one of the most abundant proteins in the HSP family. As a highly conserved molecular chaperone, HSP90 is essential for the integrity and function of numerous signaling client proteins. Activated by cellular stress, HSP90 plays a central role in myocardial I/R injury and cardioprotection during ischemic preconditioning and pharmacological conditioning [5,7,25]. Our laboratory recently found the important role of HSP90 in postconditioning [6,19,26,27]. HSP90, especially in the mitochondria, facilitates translocation of mitochondria proteins in cardioprotection against I/R-induced injury. We had earlier reported that HSP90 mediates the mitochondrial translocation of PKCε and leads to cardioprotection in IPostC [19]. Our recent study shows that HSP90-mediated mitochondrial import of Cx43 is critical during hypoxic postconditioning-induced cardioprotection [6]. Moreover, Small et al. reported that morphine reduces myocardial infarct size via mitochondrial HSP90 [8]. However, the underlying mechanism of HSP90-mediated cardioprotection by morphine is not yet fully understood. Recently, Lei et al. used a rat heart model pretreated with remifentanil and showed that the opioid-induced cardioprotection was related to activating the PI3K/Akt pathway [28]. However, whether HSP90/Akt is responsible for the cardioprotection of MP is unclear.

Akt is a serine/threonine kinase, important in the regulation of cellular activation, inflammatory responses, and apoptosis. This enzyme can be activated by growth factors, cytokines, and morphine [29]. As one of HSP90’s substrates, Akt plays a vital role in protecting the heart from I/R injury [30]. HSP90 contributes to the functional stabilization of Akt by formatting an HSP90-Akt protein complex, which phosphorylated and activated the Akt signaling. Once activated, Akt triggers a cascade of cellular signals to exert anti-apoptotic effects. However, a reduction in the HSP90/Akt binding results in Akt inactivation and cell apoptosis [31]. Therefore, in this study, we tested the links between these two signals during MP. The results showed that MP significantly increased the protein levels of HSP90 and p-Akt. By suppressing HSP90 function with GA, the MP-induced upregulation of p-Akt was inhibited, while suppressing Akt with GSK had no effect on HSP90, indicating that HSP90 is critical for the activation of Akt during MP. Therefore, our data suggest for the first time that p-Akt, downstream of HSP90 is related to MP-induced cardioprotection.

Increasing evidence shows that complement activation plays an important role in myocardial I/R injury [32]. After activation, the terminal complement components C5a and C5b-9 are deposited in the ischemic area and induce myocardial necrosis [33]. Furthermore, C5a also promotes immune responses by accumulating inflammatory cells and releasing pro-inflammatory cytokines including TNF-α, IL-1β, and IL-6, thereby causing indirect myocardial damage [15,34]. Therefore, C5a is a key mediator of myocardial I/R injury. Inhibition of C5 using anti-C5 antibodies or C5a receptor antagonists has been shown to significantly reduce myocardial I/R injury and improve outcomes in numerous *in vitro* and *in vivo* models [35,36]. There is also evidence that ischemic preconditioning attenuates myocardial I/R injury by inhibiting complement activation [16]. However, whether morphine has a similar effect as preconditioning on inhibiting the complement system had not been reported up to now. In this study, we found relatively low levels of C5a in the MP group but significantly higher levels in the I/R group, indicating the vital role of C5a in MP-mediated cardioprotection.

Recently, a link between complement activation and the NF-κB signaling pathway was discovered [37]. NF-κB is a latent gene regulatory protein that plays a paramount role in regulating inflammation and cell survival, being particularly responsive to pro-inflammatory cytokines TNF-α and IL-1 during myocardial I/R [38,39]. Previous research showed that C5a triggered inflammation by inhibiting the phosphorylation of PI3K and by activating the NF-κB pathway [37]. In addition, IPostC-induced anti-inflammation occurs via the inhibition of the NF-κB pathway [17]. However, the interaction between the complement and NF-κB in MP is unclear. This study showed for the first time that morphine simultaneously inhibited the activation of the C5a/NF-κB signaling and the release of TNF-α, IL-1β, and ICAM-1. Meanwhile, treatment with the C5a inhibitor, PMX, enhanced the MP-induced downregulation of NF-κB, while the NF-κB inhibitor QNZ did not affect C5a, indicating that the inhibition of the C5a/NF-κB signaling pathway is involved in MP-induced cardioprotection.

The role of HSP90/Akt interaction with C5a/NF-κB in MP is under investigation. HSP90 protects cells from complement-dependent cytotoxicity, while inhibition of HSP90 enhances complement-induced cell death [40]. Moreover, activating the PI3K/Akt pathway attenuates the I/R-induced inflammation by inhibiting the complement [41,42]. Recently, it was shown that phosphorylated Akt negatively regulates NF-κB signaling to limit pro-inflammatory responses [43,44]. The role of the HSP90/Akt interaction with C5a/NF-κB in MP is unclear. In this study, we found that the MP-induced C5a/NF-κB inhibition was accompanied by enhanced HSP90 and p-Akt. Both the HSP90 inhibitor (GA) and Akt inhibitor (GSK) triggered C5a/NF-κB signaling and enhanced cardiomyocyte apoptosis. To the best of our knowledge, it is the first description of the interaction between HSP90/p-Akt and C5a/NF-κB in MP. Contrary to other reports [45], our study mainly explored whether HSP90 is involved in MP, underpinning the suppressing of the C5a and NF-κB signaling. Our results suggest a possible therapeutic target in MP, which provides a reference value for clinical guidance.

This study had some limitations. First, we only established a rat *in vivo* I/R model to explore the molecular mechanism of MP cardioprotection, and we did not further study or verify the clinical relevance of our experiments. Second, in this study, we used only the HSP90-specific inhibitor GA. However, the over or under-expression of HSP90 with the use of viral vectors can be applied to further explore its role in MP in future studies. Third, given that the components of the complement system are diverse, further research is needed to explore whether HSP90 regulates the myocardial inflammatory response through other complement components, including C1, C3 etc. Moreover, the detailed regulation mechanism of HSP90 over C5a still needs to be explored.

## 5 Conclusion

This study shows that HSP90 is critical for MP-mediated cardioprotection possibly by promoting phosphorylation of Akt and inhibiting activation of C5a and NF-κB signaling and subsequent myocardial inflammation, ultimately attenuating I/R-induced infarct size and cardiomyocyte apoptosis. Our results provide new insights for MP-mediated cardioprotection against I/R injury and may identify a potential therapeutic target.
